# In Silico Study of Natural Polyphenols as Potential Metabolic Modulators in Mitigating Lipotoxicity in Non-Alcoholic Fatty Liver Disease via Thyroid Hormone Receptor Alpha Activation

**DOI:** 10.3390/cimb47090777

**Published:** 2025-09-19

**Authors:** Evangelia K. Konstantinou, Athanasios A. Panagiotopoulos, Maria Dimitriou

**Affiliations:** Department of Nutritional Science and Dietetics, School of Health Sciences, University of the Peloponnese, Antikalamos, 24100 Kalamata, Greece; e.konstantinou@go.uop.gr

**Keywords:** NAFLD, thyroid hormone receptor alpha (THRA), polyphenolic compounds, lipid metabolism, dietary polyphenols, molecular docking, fatty acid oxidation

## Abstract

Non-alcoholic fatty liver disease (NAFLD) is a metabolic disorder described by the deposition of triglycerides in the liver, which primarily occurs due to insulin resistance and obesity. Thyroid hormone receptor alpha (THRA) is involved in metabolic pathways that promote lipolysis, which can prevent the accumulation of liver fat. As a possible treatment for NAFLD, this in silico study examines the binding interactions between THRA and polyphenols and flavonoids present in fruits and vegetables. Including caffeic acid, curcumin, and chlorogenic acid, the binding affinities of the natural substances to THRA were found comparable to the hormone T3, boosting the THRA–TRAP220 complex, promoting fatty acid oxidation, while decreasing lipid accumulation in the liver.

## 1. Introduction

Non-alcoholic fatty liver disease (NAFLD) is a metabolic disorder mainly characterized by the accumulation of triglycerides in the hepatocytes [1,2,3,4,5]. Its progression is associated with insulin resistance and obesity, without the contribution of alcohol as a causative factor [6]. Meta-analysis studies indicate that NAFLD has emerged as the most common chronic liver disease worldwide, estimated to affect 55.4% of population by 2040 [7]. Although NAFLD is usually an asymptomatic disease, it is a leading cause of liver-related morbidity and mortality, commonly in men [8]. NAFLD develops into two pathological conditions; Non-Alcoholic Fatty Liver (NAFL), which is defined by steatosis as first stage of the disease and is detected in the majority of patients, and Non-Alcoholic Steatohepatitis (NASH), which means further liver damage, including inflammation and fibrosis [1,3,4]. Chronic inflammation and poor lipid metabolism brought on by obesity and insulin resistance (IR) can cause NAFLD to proceed to NASH and ultimately to cirrhosis, hepatocellular carcinoma (HCC), and mortality. Concerning statistics revealed that among patients with biopsied NAFLD, the prevalence of NASH was 59.1% worldwide [6].

According to the report of the International Consensus Panel on NAFLD/NASH published in 2023, the term NAFLD (Non-Alcoholic Fatty Liver Disease) has been replaced by the term MASLD (Metabolic Associated Steatotic Liver Disease), as the latter better reflects the metabolic dysfunction underlying the disease [9]. While we are aware of this new nomenclature, we have chosen instead to consistently refer to the condition by the term NAFLD in this manuscript. The main reason is to ensure consistency with the majority of studies cited in our study, most of which were published before 2023 and widely use the term NAFLD. In this way, the article attempts to reduce confusion for the readers in placing this work in comparison with the literature existing previously while still giving credence to the newly adopted terms.

There is compelling evidence that the selective activation of hepatic thyroid hormone receptor alpha (THRA) effectively prevents fat accumulation in the liver. This finding proposes a novel treatment strategy for NAFLD by enhancing hepatic fat oxidation [10]. THRA, encoded by the THRA gene, is a member of the nuclear receptor super-family and plays a pivotal role in mediating the biological effects of thyroid hormone metabolism [11]. In the liver and kidneys, THRA plays a key role in lipid and glucose metabolism, as well as influencing obesity and diabetes mellitus, affecting the body’s overall energy balance [12]. Studies have confirmed the association of NAFLD with other metabolic dysfunctions, such as hypothyroidism, most of which mainly regulated by the action of thyroid hormones [13]. Given the above, exploitation of thyroid hormone action and selectivity of thyroid hormone receptors has been repeatedly suggested as a treatment for various health issues [14].

THRA functions as a ligand-dependent transcription factor, activated by 3,5,3′-triiodo-L-thyronine (T3), the biologically active form of thyroid hormone, modulating the expression of genes involved in lipid biosynthesis and fatty acid oxidation [15]. The majority of THRA-associated diseases are attributed to mutations in the Ligand Binding Domain (LBD) of THRA, which impair the binding of T3 [16]. When T3 binds to THRA, the expression of genes involved in fatty acid β-oxidation is increased, while genes involved in lipogenesis and triglyceride deposition in the liver are suppressed. Low T3 levels cause an increase in lipogenesis in the liver, which may aid in the development of NAFLD [13]. T3 induces the interaction of THRA with Thyroid Hormone Receptor–Associated Protein 220 (TRAP220), a key protein in lipolysis. The THRA/TRAP220 complex plays a pivotal role in activating the transcription of genes involved in lipolysis, a critical process for reducing fat accumulation in the liver [17]. Activating this pathway can help reverse NAFLD by decreasing hepatic lipid content and enhancing overall metabolic health. For individuals with impaired T3 production, natural product-based dietary compounds may serve as an alternative to enhance or restore proper thyroid receptor function. These natural compounds that bind to THRA offer a safer and potentially more effective treatment option for NAFLD, with a lower risk of side effects. Despite the potential therapeutic potential, research exploring the role of natural products in modulating the THRA/TRAP220 complex remains limited. Therefore, this study aims to investigate natural polyphenolic compounds—many of which are abundant in peaches—as potential ligands for THRA and to evaluate the formation of the THRA/TRAP220 complex, presenting lipolytic properties through the above mechanism.

## 2. Materials and Methods

The structure of THRA receptor was extracted from AlphaFold database (https://alphafold.ebi.ac.uk/, accessed on 15 September 2025) [18] (Code: AF-P10827-F1) in the pdb format and was introduced in the GalaxyWEB server (https://galaxy.seoklab.org/, accessed on 15 September 2025) [19], together with the agonistic molecule T3 and the natural products for blind docking without specific grid box. The most favorable solutions (with the highest binding affinity) were analyzed using the PyMOL V2.4 program (https://www.schrodinger.com/, accessed on 15 September 2025), focusing on amino acids that were less than 4.5 Å from any atom of the ligand. The liganded THRA conformation was introduced to GalaxyWater-wKGB server (https://galaxy.seoklab.org/cgi-bin/submit.cgi?type=WKGB, accessed on 15 September 2025) [20], leading to corresponding complexes, including water molecules. In PyMOL, hydrogen bonds have been identified to occur either with or without co-crystalized water molecules.

Polyphenolic structures and T3 in the canonical SMILES format were extracted from the PubChem database (https://pubchem.ncbi.nlm.nih.gov/, accessed on 15 September 2025) [21] and converted to pdb files through Open Babel (http://openbabel.org, accessed on 15 September 2025) [22]. Following the complex’s final refinement, fully flexible ligand–receptor binding was performed using the particular application Galaxy7TM on the GalaxyWeb server (https://galaxy.seoklab.org/cgi-bin/submit.cgi?type=7TM, accessed on 15 September 2025) [23]. The UCSF Chimera program (https://www.cgl.ucsf.edu/chimera/, accessed on 15 September 2025) [24] was used to visualize the ligand-receptor final complex in the pdb format. THRA–ligand complexes were introduced in the HEX 8.0.0 program (http://hex.loria.fr/, accessed on 15 September 2025) [25] with the identified structured TRAP220 molecule (generated by AlphaFold) in the pdb format for protein–protein docking. Program output provides the optimal solution along with the matching ΔG.

To ensure the reliability of our docking protocol, and based on modern guides for reliable docking methods [26], a validation study was performed in which predicted with the GalaxyWEB algorithm ligand binding poses in docking were compared with experimental THRA–ligand complex structures determined through experimental techniques. In specific, crystal structures (PDB IDs: 1NAV, 2H77, 2H79, 3HZF, 3ILZ, 3JZB, 4LNW, 4LNX, 7QDT) were acquired through UniProt (https://www.uniprot.org/uniprotkb/P10827, accessed on 15 September 2025), providing a structural benchmark for our analysis.

For the docking analysis, the GalaxyWeb software was used, taking the corresponding receptor sequence derived from relevant structures and replacing the natural ligand with the corresponding counterpart in each structure. Specifically, the sequence of each crystal structure was imported into GalaxyTBM (https://galaxy.seoklab.org/cgi-bin/submit.cgi?type=TBM, accessed on 15 September 2025) in order to create the structure of each receptor [27]. Using Open Babel (http://openbabel.org, accessed on 15 September 2025) [22], the construction of the pdb files of the corresponding ligands was carried out. Then, using Galaxy7TM on the GalaxyWEB server (https://galaxy.seoklab.org/cgi-bin/submit.cgi?type=7TM, accessed on 15 September 2025) [23] the fully flexible docking of the receptors with the corresponding ligands was carried out.

The UCSF Chimera program (https://www.cgl.ucsf.edu/chimera/, accessed on 15 September 2025) [24] was used to compare the crystal structures with the corresponding first (model 1 with highest binding affinity) solutions from the docking algorithm. Using such an analysis, we could assess whether our docking algorithm could accurately reproduce the ligand orientation upon binding for known ligands in the THRA binding pocket.

The online program SwissDock (https://www.swissdock.ch/, accessed on 15 September 2025) [28,29], which is based on the Autodock Vina algorithm [30], and the Glide program of the Schrödinger suite (https://www.schrodinger.com/platform/products/glide/, accessed on 15 September 2025) were used to confirm the binding of the ligands to the THRA receptor. The online program pyDockWEB (https://life.bsc.es/pid/pydockweb, accessed on 15 September 2025) [31] was used to confirm the binding of the THRA–ligand complex to the TRAP220 receptor.

Prime program of the Schrödinger suite (https://www.schrodinger.com/platform/products/prime/, accessed on 15 September 2025) was used to calculate and confirm the binding energy of the ligands with the receptor, following the MM/GBSA (Molecular Mechanics/Generalized Born Surface Area) methodology [32].

The online program SwissADME (www.swissadme.ch) was used to calculate the critical pharmacokinetic parameters for the compounds.

## 3. Results

Comparative analysis between docked and experimental crystal structures showed a high level of agreement, measured through low Root Mean Square Deviation (RMSD) values. The comparison of each crystal structure with the corresponding model from docking protocol is presented in Supplementary Figure S1. The RMSD values obtained for the validation ranged between 0.76 and 0.81 Å across the tested crystal structures, indicating that the docking protocol reliably reproduced experimental ligand poses. This exercise in validation confirms the effectiveness of our computational tool and validates its accuracy in predicting 21 natural compounds’ binding behavior in this study. By proving that our tool can reproduce accurately ligand binding conformation determined through experimental techniques, confidence in predictive accuracy of our docking tool for investigating potential modulators of THRA is increased.

**Figure 1:**
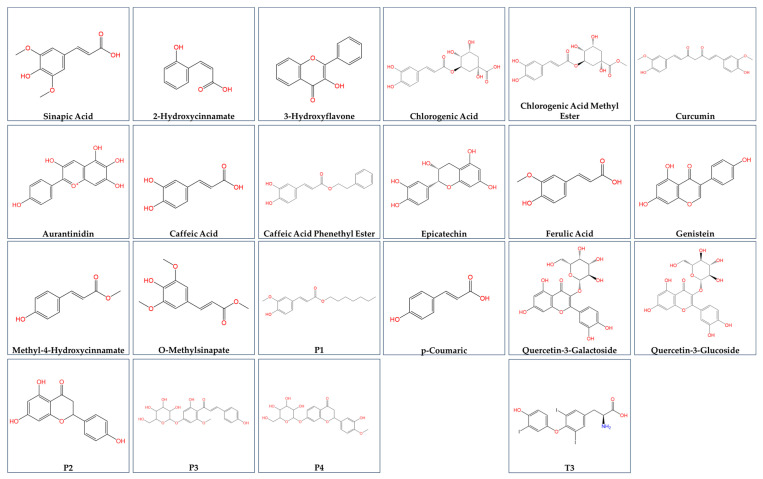
The chemical structures of natural products used in the present study.

The structures of the compounds used in the present study are presented in Figure 1. A total of 21 polyphenol compounds and T3 were studied for their effect on THRA activation.

**Figure 2:**
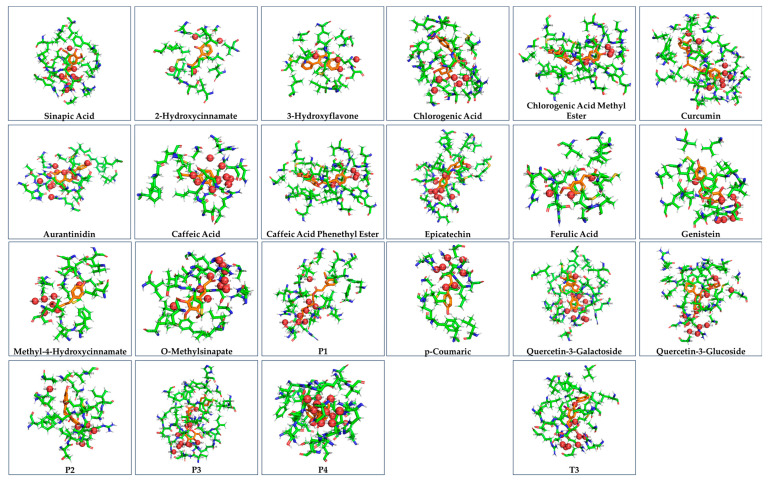
The hydrasized binding pocket of THRA receptor with the natural products.

The structures of the bound ligands in the receptor are presented in Figure 2. Green color shows the amino acids, which form strong intermolecular forces with the ligands, which are shown in orange. Red spheres show the water molecules, which participate in hydrogen bonds, enhancing the binding of the ligands to the receptor binding site (PHE_218_, ILE_222_, ALA_225_, MET_256_, MET_259_, SER_260_, ALA_263_, LEU_276_, SER_277_, LEU_292_, ILE_299_, VAL_395_, and LEU_403_).

**Figure 3:**
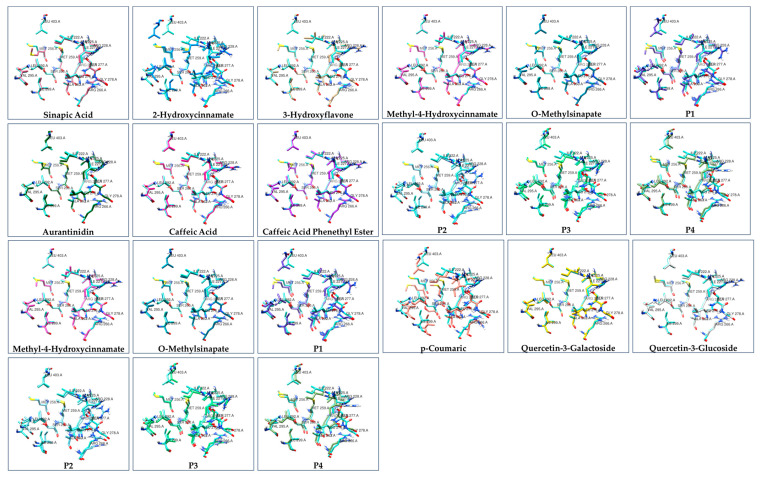
Comparison of the amino acid residues and 3D conformation in the binding groove of ΤHRA receptor after binding of the T3 (light blue) and the natural products.

It is important to note that natural compounds bind to the same amino acids that T3 binds to (PHE_218_, ILE_222_, ALA_225_, MET_256_, MET_259_, SER_260_, ALA_263_, LEU_276_, SER_277_, LEU_292_, ILE_299_, VAL_395_, and LEU_403_) (this fact was also confirmed by the docking experiments through the SwissDock and Glide programs). For this purpose, the amino acid structures in the binding pocket were compared (Figure 3). In this comparison, light blue represents the amino acids as they are found in the T3 binding site (all atoms of T3 interact strongly with the amino acid residues either through hydrogen bonds or van der Waals interactions), while different colors represent the amino acids for each compound. As shown, the compounds lead to a similar arrangement of receptor amino acids in the binding pocket region.

**Figure 4:**
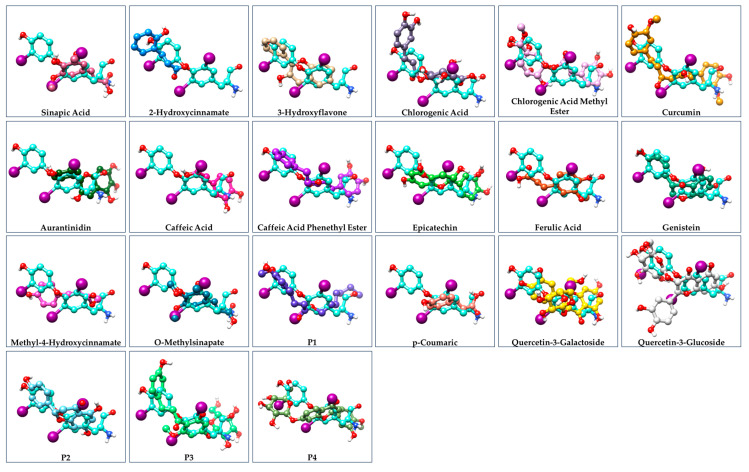
Comparison of the three-dimensional structure of T3 with the natural products used in this work. The structures have been extracted from Chimera program as they bind to the THRA binding pocket.

Ιn addition, comparing the binding positions of T3 and natural compounds is important. This analysis suggests that these compounds bind to the same region of the receptor, with their chemical groups oriented in a similar manner to that of T3. The orientation is depicted in Figure 4. In Supplementary Figures S2 and S3, the corresponding comparison of the results from SwissDock and the Glide program of the Schrödinger suite is performed. Supplementary Figure S4 compares the binding pocket of THRA with T3 (red spheres) as the ligand using GalaxyWEB, SwissDock, and Glide Docking methods.

**Figure 5:**
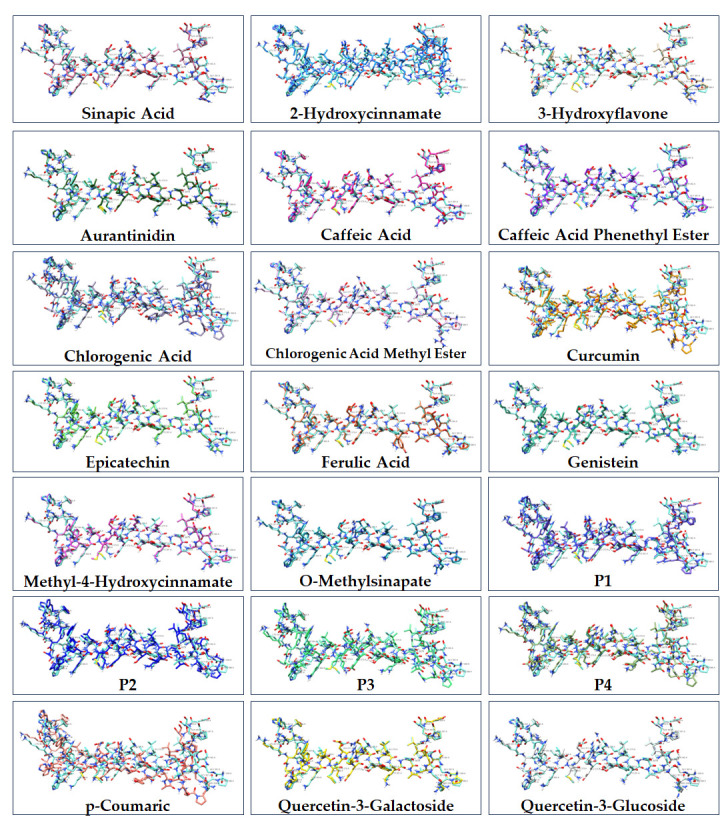
Comparison of the three-dimensional conformation of the C-Terminal region of THRA (AA 350–400) after binding of T3 and natural products in the binding pocket.

The arrangement of THRA receptor amino acids 350–400, which are located at the C-Terminal of the receptor, is critical for their binding to the TRAP220 receptor. As shown in Table 1, natural compounds bind strongly to the THRA receptor, leading to modifications in its C-Terminal region (Figure 5). The binding of different ligands leads to different modifications of this amino acid region, therefore leading to a modification of the binding capacity of TRAP220 (Table 1). In general, the compounds that led to the binding of THRA to the TRAP220 receptor with a ΔG value < −582.45 Kcal/mol (corresponding to T3; T3 causes the ΤHRA receptor to be modified, which causes it to bind to the TRAP220 receptor. Consequently, ligands with a lower ΔG have a greater influence on activating the signal for the lipolysis process) through the program HEX 8.0.0. show quite promising results for the process of reducing NAFLD. It should be noted that the absolute ΔG values reported for protein–protein docking (e.g., THRA–TRAP220 interactions) are scoring functions specific to the HEX 8.0.0 software and do not represent experimentally measured thermodynamic free energies. These values are most appropriately interpreted in a relative manner, allowing comparison of the effect of different ligands on the predicted THRA conformation and its ability to interact with TRAP220.

To provide a clearer overview of the pharmacokinetic profiles, critical parameters calculated using the application of SwissADME (www.swissadme.ch) are summarized in Table 2 (all SwissADME data are presented in Supplementary Table S3). These include molecular weight (MW), consensus lipophilicity (LogP), topological polar surface area (TPSA), and the bioavailability score, which indicate the favorable drug-like character and oral bioavailability for the investigated compounds.

## 4. Discussion

Thyroid hormone receptor alpha (THRA) plays a major role in lipid and glucose metabolism. Its activity is crucial in prevention of metabolic NAFLD [34,35,36,37,38]. THRA and NAFLD are directly related through the receptor’s activity and its interaction with coactivator protein TRAP220 (Thyroid Hormone Receptor–Associated Protein 220), a factor of the Mediator complex [39]. TRAP220 binds to activated THRA and acts as a transcriptional coactivator, promoting expression of genes regulating fatty acid oxidation, mitochondrial function, and lipid transport [40]. This balance between the production and breakdown of fatty acids in the liver prevents lipid accumulation in the liver, which is a hallmark of NAFLD [40]. The THRA–TRAP220 complex that is formed leads to upregulation of enzymes involved in fatty acid degradation, such as carnitine palmitoyltransferase (CPT1), and activation of genes involved in triglyceride clearance and lipid export from the liver [40]. Through fatty acid β-oxidation in the mitochondria and downregulation of lipid synthesis, progression of NAFLD is prevented [40].

Natural products, especially flavonoids, provide a promising alternative or complementary treatment for NAFLD [41,42,43,44,45,46]. Numerous fruits and vegetables contain these polyphenolic chemicals, which are essential for controlling liver function and lipid metabolism [42,46]. Having a low caloric value, fruits and vegetables are an excellent source of vitamins, flavonoids and polyphenols, which act synergistically, enhancing metabolic pathways such as antioxidant protection, energy regulation, detoxification, inflammatory response, and hormonal balance [42,46]. Additionally, the significant amount of water in these foods facilitates the body’s absorption of water-soluble nutrients, a process aided by plant fibers [41,42,43,44,45,46]. Flavonoids are already known for their antioxidant and anti-inflammatory effects; moreover, they have been shown to influence the function of nuclear receptors, including THRA, activating fatty acid β-oxidation pathways and preventing the buildup of triglycerides in the liver [47,48,49]. The intake of the above nutrients through nutrition is a completely harmless solution but also an effective way to maximize their absorption and exploitation by the human body against many metabolic disorders, including NAFLD [48]. A group of natural products is suggested in this study as potential anti-NAFLD natural products, and THRA has been identified as one of their targets.

Peaches (*Prunus persica*) are one of the most important cash crops to fruit growers around the world [50]. They are widely cultivated, with global production exceeding 25 million tons per year, mainly from countries such as China, Italy, Spain, and Greece. They have been tracked back over 8000 years, and 2000 varieties have been recorded, due to juicy flesh, rich aroma, and high nutritional value [51,52]. Greater abundance in antioxidants and vitamins is found in peach varieties with intense yellow or deep red flesh color. Studies have shown that peaches display a multitude of properties that promote proper health of the body, particularly supporting metabolism [53,54,55]. Vitamins A, C, and E, which are essential for boosting immunity and dietary fiber, are beneficial components of a balanced diet that peaches contain [54,55]. Peaches represent one of several dietary sources rich in flavonoids and phenolic acids. Although these compounds are widely distributed in many fruits and vegetables, peaches have been studied extensively and provide a representative dietary source for these bioactives [56]. This study examines the protective impact of peaches against non-alcoholic fatty liver disease (NAFLD), alleviating THRA’s action.

From a biochemical perspective, nutrients of peach can influence lipid metabolism and inhibit lipotoxicity by modulating key cellular pathways that regulate fat storage and breakdown, including those regulated by THRA. Methyl 4-hydroxycinnamate, p-Coumaric acid, and 2-hydroxycinnamate, as dietary polyphenols with low toxicity, have been proposed as potential drugs for treatment of NAFLD [57]. They enhance the down-regulation of fat accumulation in the liver by activating peroxisome proliferator-activated receptors (PPARs), which work in conjunction with THRA to regulate fatty acid oxidation. Sinapic acid and its derivatives, present in grains and fruits, exhibit strong antioxidant effects, improving mitochondrial function and supporting THRA’s role in enhancing lipolysis [58]. O-methylsinapate exhibit great antioxidant activity which indirectly supports THRA’s role in lipid metabolism [59]. Chlorogenic acid methyl ester, widely distributed in fruits and many peach species, influences the action of THRA leading to lipolysis by activating AMP-activated protein kinase (AMPK), thereby increasing fatty acid breakdown [60]. Ferulic acid is involved in the HSL/perilipin cascade showing great antilipogenetic activity, identified as a key molecule in the amelioration of lipometabolism [61]. Caffeic acid treatment in mouse models after receiving a high-fat diet (HFD) for 8 weeks showed it as a deterrent factor in dysregulation of gene expression linked to lipid metabolism [62]. Caffeic acid phenethyl ester and chlorogenic acid also influence lipid metabolism by reducing fat accumulation and promoting THRA-related pathways for fat oxidation. In vivo studies in mice have shown effects of chlorogenic acid in fat reduction, simultaneously lowering triglyceride in the liver and cholesterol levels in plasma [63]. Curcumin I and curcumin decrease the synthesis of triglycerides in liver by inhibiting the enzyme HMG COA reductase. Also, these molecules are strong activators of AMPK, enhancing THRA-mediated fatty acid oxidation [64]. 3-Hydroxyflavone and aurantinidin improve fatty acid breakdown through ameliorating inflammation and have been found to be effective on non-alcoholic fatty liver diseases by modulating enzymes linked to THRA [65]. Epicatechin mediates fat breakdown by improving THRA-regulated mitochondrial biogenesis [66]. Natural modification of peach flavonoids such as quercetin leads to bioactive derivatives through glycosylation (quercetin-3-glucoside), establishing new pharmacological activities against liver cancer and NAFLD [67]. Genistein, an isoflavone that excerpts anti-obesity effects, increases the effect of THRA in lipid metabolism through simultaneous interaction with other proteins, such as AMPK, decreasing triacyl glyceride and cholesterol levels in the liver [68]. The present article explores the anti-NAFLD character of these compounds through metabolic pathways involving THRA.

All compounds presented in this study showed an interesting pharmacokinetic character. Using the online resource SwissADME (www.swissadme.ch), we further calculated some parameters useful for the drugability of the natural compound (Table 2 and Supplementary Table S3). Above natural products have positive pharmacokinetics and thus have a high potential for development in drugs. Specifically, molecular weight is at a level allowing for proper absorption and distribution in an organism, and balanced lipophilicity permits membrane permeability with no solubility restriction. In addition, most compounds follow simple pharmacologic evaluation rules, such as Lipinski, Veber, and Egan, and thus have high bioavailability and a potential for successful administration through an oral route [69]. It is worth noting that several of them have no, or at least minimal, toxicologic warnings (PAINS, Brenk alerts), and thus have a low potential for a toxic reaction and a high potential for success in both preclinical and clinical trials [69]. Overall, information confirms that these compounds have the potential for becoming high-potential candidates for development in drugs [69].

It has been found that there is a well-documented binding site for a second T3 molecule in the THRA receptor [70]. The amino acid residues involved in the second binding are amino acids 368–376. According to the findings of our study, TRAP220 binds to amino acid residues 350–400. However, although we performed free docking, the 21 natural compounds as well as the T3 hormone did not bind to the second site, suggesting that other factors may be required for the natural binding (presence of many water molecules). In addition, it is very likely that the binding of TRAP220 due to its high binding affinity to the THRA receptor competes for the second binding site. In vitro experiments could investigate this possibility.

An important contribution of this work lies in the novel integration of different computational layers to study the THRA pathway. While existing research proposed the polyphenol modulation of nuclear receptors previously, the current work specifically highlights their role in support of the THRA–TRAP220 interaction as a molecular event that enables lipolysis. To our knowledge, this work provides the first docking analysis to comprehensively study peach polyphenols in this context. Furthermore, by combining receptor–ligand docking, protein–protein docking, validation against experimental crystal structures, and pharmacokinetic profiling, we provide a comprehensive in silico structure for facilitating the reliability of predictions. These factors in combination overcome earlier docking-focused speculations and open up novel possibilities for experimental validation in the context of THRA-targeted nutraceuticals for NAFLD.

A critical aspect in considering THRA as a therapeutic target is the selectivity of the binding of the ligands. While the results in this study indicate that the polyphenols investigated possess a similarity to T3 in binding to THRA, the overall activation of thyroid receptors may also be influenced by Thyroid Hormone Receptor Beta (THRB) or by other nuclear receptors such as Peroxisome Proliferator-Activated Receptor alpha (PPARα) and gamma (PPARγ), leading to off-target metabolic effects [14,35,71,72]. The activation of THRB was associated with increased cardiac workload and alterations in the metabolism of cholesterol, which may not always be desirable in NAFLD patients [73]. Therefore, achieving high THRA selectivity is essential to minimize adverse events such as tachycardia, bone loss, or hypermetabolic symptoms that accompany generalized thyroid hormone excess. Future in vivo studies should delineate the therapeutic window in whole animals, defining the doses that achieve the maximal clearance of lipids in the hepatocytes, while limiting the systemic thyromimetic impacts [35]. It will be important in the safe translation of natural THRA modulators for the clinical application in NAFLD.

A further limitation of our study is that the predicted results are determined by ligand concentrations in the receptor site and may not be attained through conventional diet-based ingestion. Molecules such as quercetin or chlorogenic acid are rapidly metabolized and possess limited in vivo bioavailability. The needed hepatic levels to elicit considerable THRA modulation are therefore likely to be attained through diet alone. This highlights the need for future studies exploring optimized formulations, delivery systems, or synthetic analogs with improved pharmacokinetic properties to enable potential therapeutic applications.

## 5. Conclusions

The results of the present study demonstrate that several natural polyphenols, particularly those abundant in fruits such as peaches, display favorable predicted binding affinity for THRA and promote its interaction with TRAP220 in silico. These findings provide a computational framework for screening candidate molecules for future experimental validation. While our data support the hypothesis that modification of THRA by natural products could contribute to the reduction in hepatic lipid accumulation, these results remain predictive and need to be confirmed through in vitro assays, in vivo models, and ultimately clinical studies before any therapeutic application can be established. Therefore, the present work should be considered as a starting point for further mechanistic investigations on dietary or pharmacological interventions targeting THRA for NAFLD.

## Figures and Tables

**Table 1:** The binding strength in kcal/mol of each compound to THRA receptor and the binding strength in kcal/mol of the THRA-Ligand complex to TRAP220 receptor. * Natural products as mentioned in the work of Allam et al. in 2020 [33].

Ligand	ΔG of Binding to THRA (kcal/mol)	MMGBSA ΔG Binding Energy (kcal/mol) of GalaxyWEB Model	ΔG of THRA-Liganded Binding to TRAP220 (kcal/mol)
p-Coumaric	−7.948	−7.381	−812.60
P4 *	−17.269	−17.953	−694.70
2-Hydroxycinnamate	−5.804	−6.201	−669.50
P2 *	−12.110	−11.995	−665.11
P1 *	−10.579	−11.003	−654.50
Caffeic Acid	−8.499	−8.421	−651.59
Curcumin	−15.491	−16.113	−649.71
Methyl-4-Hydroxycinnamate	−7.914	−8.004	−641.87
Chlorogenic Acid	−13.616	−13.412	−627.93
Chlorogenic Acid Methyl Ester	−15.155	−14.950	−617.52
Quercetin-3-Galactoside	−17.321	−17.117	−599.31
Epicatechin	−11.952	−12.021	−596.21
Genistein	−9.671	−10.423	−595.28
O-Methylsinapate	−9.628	−9.527	−588.62
T3	−13.388	−13.216	−582.45
Quercetin-3-Glucoside	−15.370	−15.444	−576.29
3-Hydroxyflavone	−9.231	−9.785	−570.24
Ferulic Acid	−8.980	−9.075	−563.35
Caffeic Acid Phenethyl Ester	−12.300	−11.889	−562.89
Sinapic Acid	−9.274	−10.004	−559.81
Aurantinidin	−14.221	−12.925	−559.73
P3 *	−18.465	−17.563	−525.58

**Table 2:** Critical pharmacokinetic profiles for the investigated compounds calculated with SwissADME. * Natural products as mentioned in the work of Allam et al. in 2020 [33].

Ligand	ΜW (g/mol)	TPSA (Å^2^)	Consensus LogP	Bioavailability Score
p-Coumaric	164.16	57.53	1.26	0.85
P4 *	448.42	155.14	0.28	0.55
2-Hydroxycinnamate	163.15	60.36	1.49	0.85
P2 *	272.25	86.99	1.84	0.55
P1 *	292.37	55.76	3.83	0.55
Caffeic Acid	180.16	77.76	0.93	0.56
Curcumin	368.38	93.06	3.03	0.55
Methyl-4-Hydroxycinnamate	178.18	46.53	1.81	0.55
Chlorogenic Acid	354.31	164.75	−0.39	0.11
Chlorogenic Acid Methyl Ester	368.34	153.75	0.00	0.55
Quercetin-3-Galactoside	464.38	210.51	−0.38	0.17
Epicatechin	290.27	110.38	0.85	0.55
Genistein	270.24	90.90	2.04	0.55
O-Methylsinapate	238.24	64.99	1.75	0.55
T3	650.97	92.78	2.93	0.55
Quercetin-3-Glucoside	463.37	213.34	−0.23	0.11
3-Hydroxyflavone	238.24	50.44	2.84	0.55
Ferulic Acid	194.18	66.76	1.36	0.85
Caffeic Acid Phenethyl Ester	284.31	66.76	3.09	0.55
Sinapic Acid	224.21	75.99	1.31	0.56
Aurantinidin	287.24	114.29	0.53	0.55
P3 *	448.42	166.14	0.42	0.55

## Data Availability

Data is contained within the article or Supplementary Material.

## Supplementary Materials

The following supporting information can be downloaded at: https://www.mdpi.com/article/10.3390/cimb47090777/s1.
