# Supplementary material for: In Silico Study of Natural Polyphenols as Potential Metabolic Modulators in Mitigating Lipotoxicity in Non-Alcoholic Fatty Liver Disease via Thyroid Hormone Receptor Alpha Activation
Source: Curr Issues Mol Biol. 2025 Sep 19;47(9):777. doi: 10.3390/cimb47090777 (PMC12468250; doi:10.3390/cimb47090777)
Supplement: Supplementary file 1 [file cimb-47-00777-s001.zip › Supplementary_Information.pdf]

In silico study of natural polyphenols as potential metabolic modulators in mitigating lipotoxicity in Non-Alcoholic Fatty Liver Disease via Thyroid Hormone Receptor Alpha activation

Evangelia K. Konstantinou, Athanasios P. Panagiotopoulos, and Maria Dimitriou

Supplementary Material

Table of contents

Supplementary Figure S1........2
Supplementary Figure S2........3
Supplementary Figure S3........4
Supplementary Figure S4........5
Supplementary Table S1........6
Supplementary Table S2........7
Supplementary Table S3........10

Supplementary Figure S1

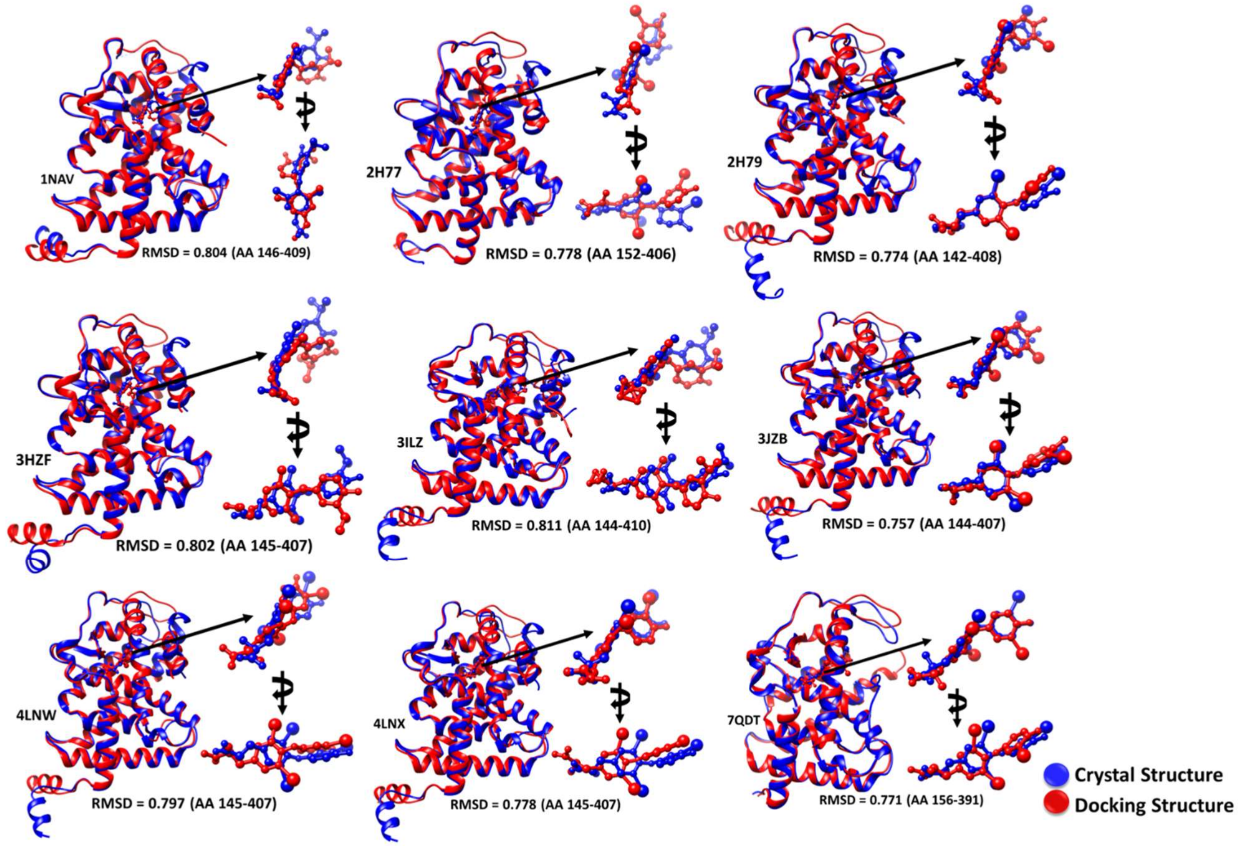

Comparison of the crystal structures of Human Thyroid Receptor alpha (THRA) with the corresponding structures resulting from the use of the docking methodology. The respective crystal is shown in blue while its predicted docking structure is shown in red. The ligands are shown with ball sticks. RMSDs refer to the pruned atom pairs from the amino acids (AA).

Supplementary Figure S2

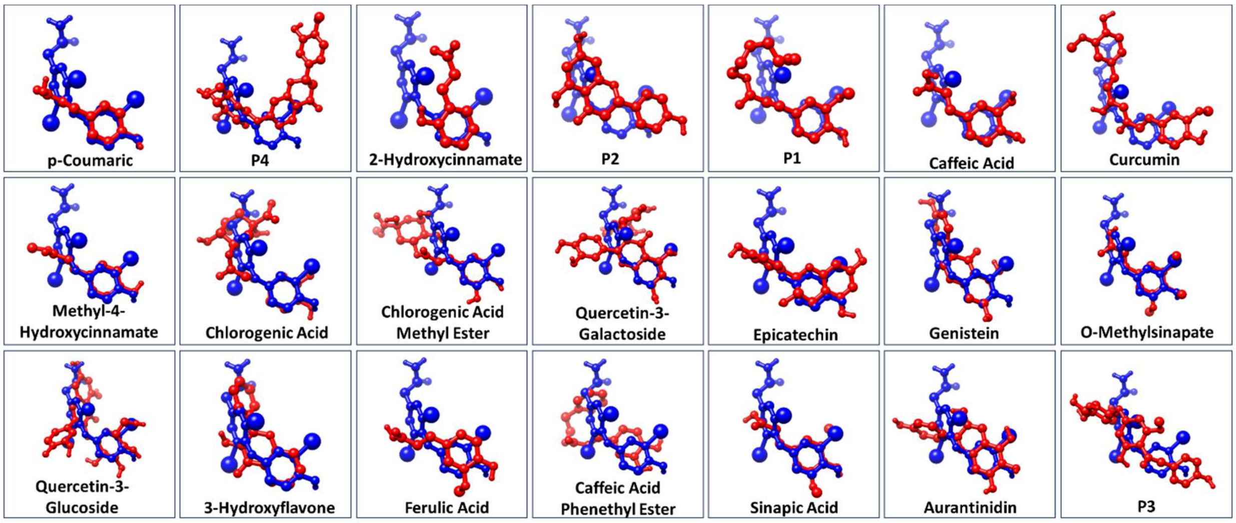

Comparison of the three-dimensional structure of T3 (blue color) with the natural products (red color) used in this work. The structures have been extracted from Chimera programm as they bind to the THRA binding pocket using the SwissDock docking program.

Supplementary Figure S3

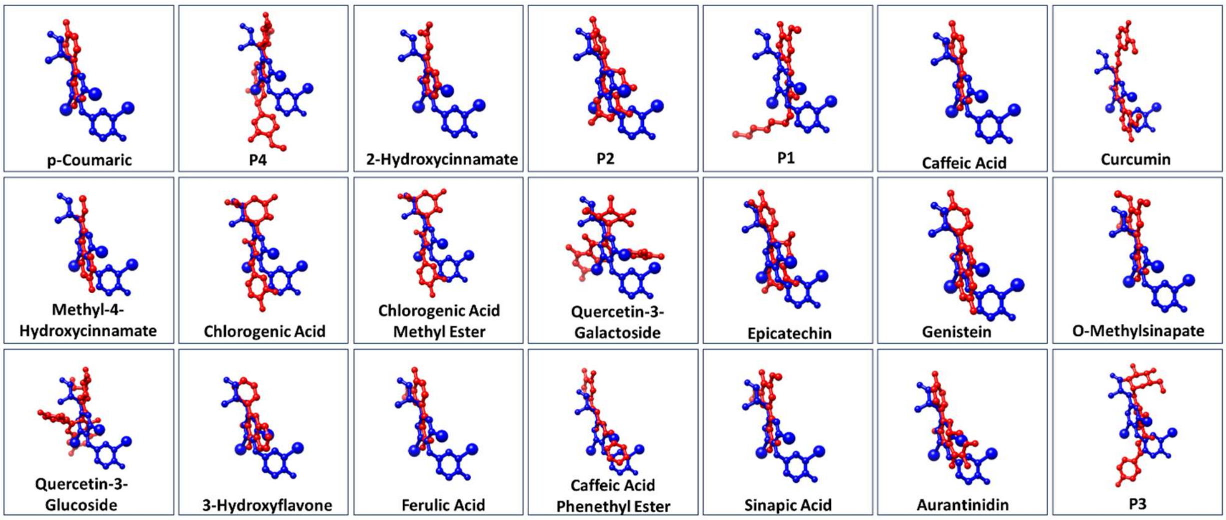

Comparison of the three-dimensional structure of T3 (blue color) with the natural products (red color) used in this work. The structures have been extracted from Chimera programm as they bind to the THRA binding pocket using the Glide docking program.

Supplementary Figure S4

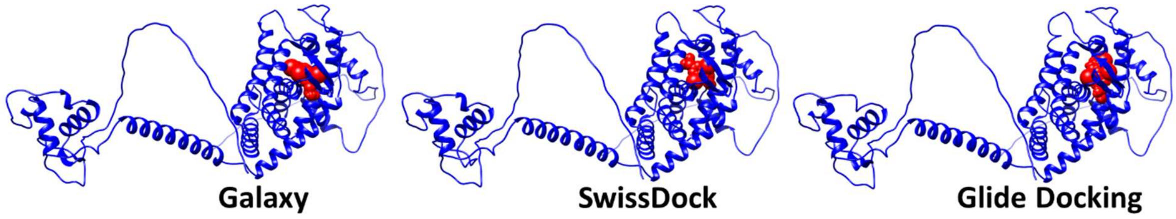

Comparison of the binding pocket of THRA with T3 (red spheres) as ligand using GalaxyWEB, SwissDock and Glide Docking methods.

Supplementary Table S1

The binding strength in terms of docking score (SwissDock and Glide Docking) and kcal/mol using the MM/GBSA method of each compound to THRA receptor.

Ligand	Binding to THRA (SwissDock Score)	Binding to THRA (Glide Docking Score)	MM/GBSA ΔG Binding Energy (Kcal/mol) of SwissDock model	MM/GBSA ΔG Binding Energy (Kcal/mol) of Glide Docking model
p-Coumaric	-6.456	-5.843	-6.326	-6.062
P4	-6.789	-6.425	-7.661	-7.475
2-Hydroxycinnamate	-7.548	-6.143	-8.026	-8.166
P2	-8.815	-9.287	-8.998	-8.932
P1	-6.948	-5.597	-6.016	-6.454
Caffeic Acid	-7.766	-5.817	-7.807	-7.024
Curcumin	-7.563	-9.794	-8.114	-8.553
Methyl-4-Hydroxycinnamate	-7.679	-7.288	-8.319	-8.276
Chlorogenic Acid	-7.410	-9.158	-9.736	-9.654
Chlorogenic Acid Methyl Ester	-8.171	-9.365	-9.856	-9.998
Quercetin-3-Galactoside	-6.895	-7.348	-5.484	-5.377
Epicatechin	-10.054	-10.072	-10.340	-10.429
Genistein	-10.018	-9.806	-11.620	-10.905
O-Methylsinapate	-6.671	-6.605	-6.154	-6.478
T3	-8.251	-8.337	-8.541	-8.586
Quercetin-3-Glucoside	-6.987	-7.988	-6.163	-6.999
3-Hydroxyflavone	-9.000	-8.463	-10.458	-10.575
Ferulic Acid	-8.239	-8.802	-7.465	-8.025
Caffeic Acid Phenethyl Ester	-8.632	-8.372	-10.854	-10.223
Sinapic Acid	-6.230	-6.662	-6.170	-6.721
Aurantinidin	-9.924	-9.268	-10.187	-10.282
P3	-11.960	-10.460	-10.406	-10.772

Supplementary Table S2

The binding affinity in terms of docking score (pyDock) of the THRA-Ligand complex to TRAP220 receptor.

**Ligand**	**Electrostatics**	**Desolvation**	**VdW**	**Total**	**Diagram of Frequency *vs* scoring energy of pyDock**
p-Coumaric	-20.900	-26.788	110.933	-36.595	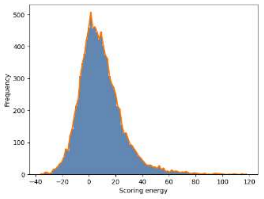
P4	-40.494	-3.454	12.764	-42.672	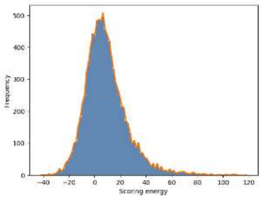
2-Hydroxycinnamate	-7.541	-38.556	34.368	-42.660	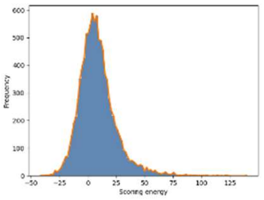
P2	-25.606	-19.189	4.020	-44.393	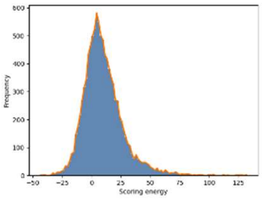
P1	-18.851	-34.176	53.772	-47.649	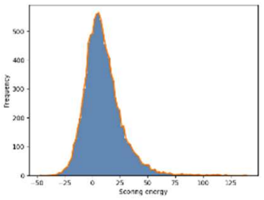
Caffeic Acid	-7.706	-38.460	45.068	-41.659	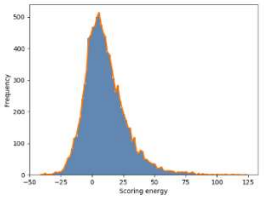
Curcumin	-11.956	-29.918	59.418	-35.933	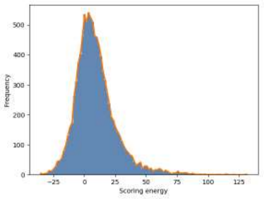

Methyl-4-Hydroxycinnamate	-12.088	-31.371	32.353	-40.223	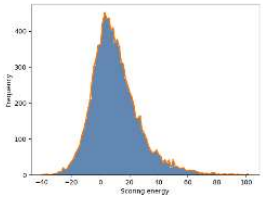
Chlorogenic Acid	-21.856	-17.621	12.296	-38.247	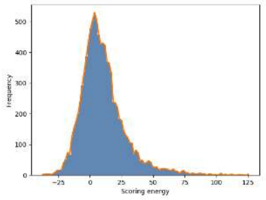
Chlorogenic Acid Methyl Ester	-32.205	-16.102	14.022	-46.904	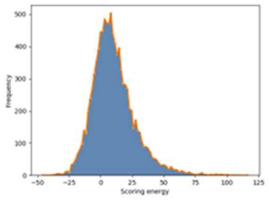
Quercetin-3-Galactoside	-18.959	-28.667	47.100	-42.916	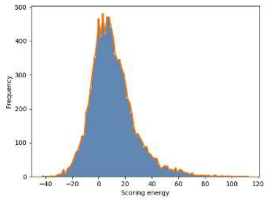
Epicatechin	-8.313	-37.107	77.087	-37.711	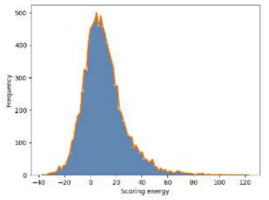
Genistein	-10.851	-33.737	18.722	-42.716	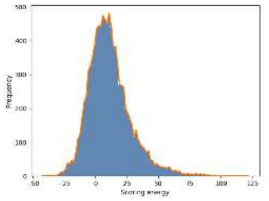
O-Methylsinapate	-19.244	-18.202	56.311	-31.815	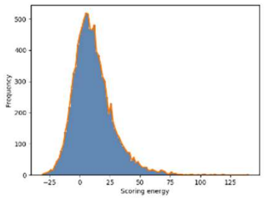
T3	-17.651	-19.253	59.111	-30.992	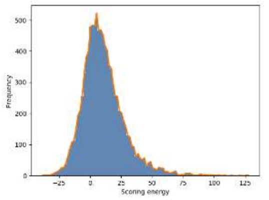

Quercetin-3-Glucoside	-20.889	-12.062	39.729	-28.979	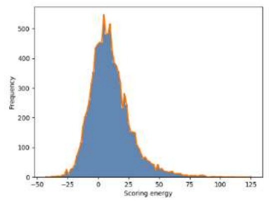
3-Hydroxyflavone	-14.024	-23.439	56.162	-31.847	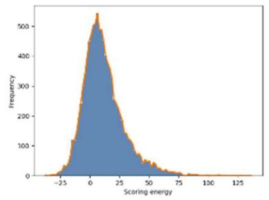
Ferulic Acid	-23.215	-10.575	36.526	-30.137	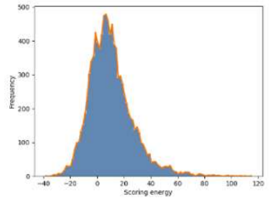
Caffeic Acid Phenethyl Ester	-6.888	-35.537	38.989	-38.526	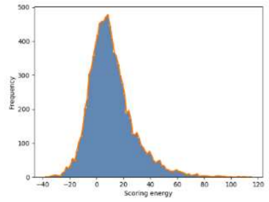
Sinapic Acid	-35.958	1.602	52.564	-29.100	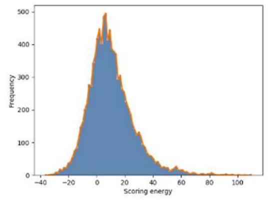
Aurantinidin	-15.630	-29.301	176.484	-27.283	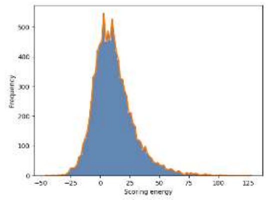
P3	-17.057	-10.702	-14.578	-29.217	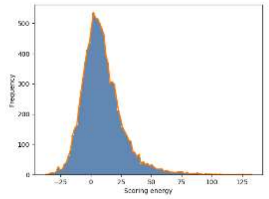

Supplementary Table S3

Please, refer to the Supplementary Table S3.xlsx Excel file.

Absorption, Distribution, Metabolism and Excretion (ADME) values, calculated with the online resource www.swissadme.ch. See the resource's help and the main text for further details and corresponding references.
